# Quorum-quenching enzyme Est816 assisted antibiotics against periodontitis induced by *Aggregatibacter actinomycetemcomitans* in rats

**DOI:** 10.3389/fcimb.2024.1368684

**Published:** 2024-05-08

**Authors:** Junmin Wang, Tianjuan Ju, Lifeng Guo, Wenwen Shan, Qianxia Wu, Haichuan Zhang, Jing Zhang

**Affiliations:** ^1^ Stomatological Hospital and College, Key Lab. of Oral Diseases Research of Anhui Province, Anhui Medical University, Hefei, Anhui, China; ^2^ State Key Laboratory of Oral & Maxillofacial Reconstruction and Regeneration, National Clinical Research Center for Oral Diseases, Shaanxi Clinical Research Center for Oral Diseases, Department of Pediatric Dentistry, School of Stomatology, The Fourth Military Medical University, XI''an, Shaanxi, China

**Keywords:** *N*-acylhomoserine lactonases, biofilm, antibiotic, periodontitis, *Aggregatibacter actinomycetemcomitans*

## Abstract

**Introduction:**

Quorum-quenching enzyme Est816 hydrolyzes the lactone rings of *N*-acyl homoserine lactones, effectively blocking the biofilm formation and development of Gram-negative bacteria. However, its applications in the oral field is limited. This study aimed to evaluate the efficacy of enzyme Est816 in combination with antibiotics against periodontitis induced by *Aggregatibacter actinomycetemcomitans in vitro* and *in vivo*.

**Methods:**

The antimicrobial efficacy of enzyme Est816 in combination with minocycline, metronidazole, and amoxicillin was determined using the minimum inhibitory concentration test. The anti-biofilm effect of enzyme Est816 was assessed using scanning electron microscopy, live/dead bacterial staining, crystal violet staining, and real-time quantitative PCR. Biocompatibility of enzyme Est816 was assessed in human gingival fibroblasts (HGF) by staining. A rat model of periodontitis was established to evaluate the effect of enzyme Est816 combined with minocycline using micro-computed tomography and histological staining.

**Results:**

Compared to minocycline, metronidazole, and amoxicillin treatment alone, simultaneous treatment with enzyme Est816 increased the sensitivity of biofilm bacteria to antibiotics. Enzyme Est816 with minocycline exhibited the highest rate of biofilm clearance and high biocompatibility. Moreover, the combination of enzyme Est816 with antibiotics improved the antibiofilm effects of the antibiotics synergistically, reducing the expression of the virulence factor leukotoxin gene (*ltxA*) and fimbria-associated gene (*rcpA*). Likewise, the combination of enzyme Est816 with minocycline exhibited a remarkable inhibitory effect on bone resorption and inflammation damage in a rat model of periodontitis.

**Discussion:**

The combination of enzyme Est816 with antibiotics represents a prospective anti-biofilm strategy with the potential to treat periodontitis.

## Introduction

Periodontitis is a chronic multifactorial inflammatory disease characterized by progressive loss of tooth-supporting apparatus and is associated with dysbiotic plaque biofilms (Slots, 2017). Aggressive periodontitis describes a set of unusual, typically severe, and fast-progressing types of periodontitis that are particularly common in young patients (Teughels et al., 2014). *Aggregatibacter actinomycetemcomitans* (*A. actinomycetemcomitans*) is the most commonly found species in aggressive periodontitis (Åberg et al., 2015). Given its high incidence and the potential to cause loss of teeth, periodontitis poses a significant public health concern.

Biofilms with extracellular polymeric matrix compounds are highly resistant to conventional antimicrobials and interact with the host immune system to cause inflammation and disease. This physiological state persists during active and resting periods until the microbial biofilm is effectively removed, and inflammation eventually subsides (Mah and O’Toole, 2001). Mechanical debridement and antibiotic therapy are the primary treatment strategies for periodontitis. However, it is extremely difficult to eradicate biofilms completely (Feres et al., 2018). Following this treatment, a small number of planktonic *A. actinomycetemcomitans* cells readily reaggregate and form new biofilms, leading to recurrent periodontitis (Kaplan, 2010). Additionally long-term exposure to systemic antibiotics increases the risk of bacterial resistance (Tseng et al., 2011). It is more feasible to inhibit the formation of biofilms than to break down pre-formed biofilms, which are usually characterized by dense three-dimensional structures and intricate antibiotic resistance mechanisms. Therefore, utilizing biofilm inhibitors in combination with antibiotics holds promise in inhibiting biofilm formation and enhancing antimicrobial activity.

The quorum quenching system inhibits biofilm formation and maturation by regulating the expression of virulence factors in a density-dependent manner (Postat and Bousso, 2019). The dominant signaling molecules associated with the quorum sensing (QS) system in Gram-negative bacteria are *N*-acyl homoserine lactones (AHLs) (Geske et al., 2008; Muras et al., 2022). AHL lactonases target and hydrolyze the lactone ring of AHLs, thereby disrupting AHL-mediated QS during biofilm development (Billot et al., 2020). Several studies have reported that AHL lactonases can act as anti-biofilm agents by reducing the production of virulence factors and inhibiting biofilm formation in Gram-negative species such as *Pseudomonas aeruginosa* and *Porphyromonas gingivalis* (Asahi et al., 2010; Fan et al., 2017; Parga et al., 2023).

A novel AHL-degrading enzyme Est816 with high hydrolytic activity and excellent stability against all types of medium- and long-chain AHLs was cloned by a research team that performed the experiments described in the study by Fan et al (Fan et al., 2012). It was hypothesized that the combined application of AHL lactonase Est816 and antibiotics may have a synergistic effect in eradicating biofilms and thus serve as an effective alternative for the treatment of periodontitis in rats. This study is the first to evaluate the effects of applying AHL lactonases in combination with antibiotics against periodontitis induced by *Aggregatibacter actinomycetemcomitans in vitro* and *in vivo*.

## Materials and methods

### Bacterial strains and reagents

*A. actinomycetemcomitans* ATCC 43717 (Guangdong Microorganism Culture Collection Center, Guangzhou, China) was used as the representative bacterial strain. After the growth of the microorganism on a Columbia blood agar plate, a single colony was extracted by inoculation loop and cultured in brain heart infusion broth (Hopebio Qingdao, Qingdao, China) containing 5 mg/L hemin and 1 mg/L menadione for 48 hours in an anaerobic environment at 37°C and 5% CO_2_. According to the previous study, expression and purification of Est816 was successfully prepared (Fan et al., 2012). Based on our previous study findings (Zhao et al., 2024), 12 U/mL of enzyme Est816 exerted a significant anti-biofilm effect against *A. actinomycetemcomitans* with minimal effect on bacterial growth and was used in this study.

### Antimicrobial susceptibility

The minimal inhibitory concentrations (MICs) were defined as the lowest concentrations of antibiotics that resulted in no visible growth of *A. actinomycetemcomitans*. Antimicrobial susceptibility testing was performed using the broth microdilution method. 50 μL of the bacterial suspension (1× 10^6^ CFU/mL) (OD_600 = _0.1) was added to equal volume of metronidazole (MTZ), amoxicillin (AMX), and minocycline (MINO) on the 96-well plate (MedChemExpress, NJ, U.S.A.) (Lachica et al., 2019). The three antibiotics were diluted in brain heart infusion broth with final concentrations ranging from 0.125µg/mL to 128µg/mL. In the experimental groups, each group of antibiotics containing bacterial suspension was supplemented with enzyme Est816 at a final concentration of 12 U/mL. Negative and positive controls were administered with equal volumes of inactivated enzyme Est816 and PBS. The plates were incubated for 24 h at 37°C. The MICs were determined as previously described in the literature (Goodwine et al., 2019; Allkja et al., 2020). The inhibitory effects of the antibiotics in the presence of specific concentrations of enzyme Est816 (12 U/mL) were determined using spectrophotometry at 600 nm. Each experiment was repeated in triplicate. The concentrations that significantly reduced the growth and concentrations of *A. actinomycetemcomitans* without growth inhibition (sub-MIC) were selected for further experiments.

### Biofilm assessment

A suspension of *A. actinomycetemcomitans* in mid-exponential phase was diluted to OD_600 = _0.1 (1.0 × 10^6^ CFU/mL), and 500 µL were seeded onto round coverslips (14 mm diameter) on the bottom of 24-well plate, which was cultured with MTZ (sub- MIC or MIC), AMX (sub-MIC or MIC) and MINO (sub-MIC or MIC) alone or in combination with enzyme Est816 for 48 hours to form biofilms. Scanning electron microscopy (SEM) (Zhao et al., 2024) was used to investigate the biofilm morphology. *A.* actinomycetemcomitans was cultured with MTZ (sub-MIC or MIC), AMX (sub-MIC or MIC) and MINO (sub-MIC or MIC) alone or in combination with the enzyme Est816 for 48 hours to form a biofilm, subsequently washed with PBS and fixed in 2.5% (vol/vol) cold (4°C) glutaraldehyde overnight. The samples were then dehydrated using a graded ethanol series (30, 50, 70, 80, and 90%) for 15 minutes. After critical point drying and ion coating with gold (Ion Sputtering, Cressington 108Auto), SEM analysis was performed to assess the morphology of *A. actinomycetemcomitans*.

For crystal violet semi-quantitative biofilm assay, 500 μL of *A. actinomycetemcomitans* suspension (1 × 10^6^ CFU/mL) was added to 24-well plates, and 500 μL PBS, antibiotics (sub-MIC or MIC), and enzyme Est816 were added to each well individually to co-culture with the biofilm. After incubation for 48 hours at 37 °C under microaerophilic conditions, the biofilm was fixed with 400 μL *paraformaldehyde* solution for 15 minutes. The solution was then removed, and the biofilms were stained with crystal violet solution (0.1%, 300 μL) for 20 minutes. Next, the biofilms were washed thrice, and the crystal violet stain was dissolved with 400 μL of 95% ethanol. The optical density values of the semi-quantitative analysis of the biofilm were detected at 570 nm using a microplate reader. To determine biofilm reduction (Shakya et al., 2022), the percentages of biofilm was calculated as follows: $Biofilm\;Reduction\%=\frac{\text{Abs Control }-\text{ Abs Sample}}{\text{Abs Control }}\;\times \;100\%$ (Abs Control represented the group without the addition of drugs). For testing biofilm eradication rates, *A. actinomycetemcomitans* suspension in the 96-well plate was incubated at 37^◦^C for 48 hours to form a mature biofilm. Then, the antibiotics at concentrations of sub-MIC, MIC with or without enzyme Est816 were added to disassemble mature biofilm for another 24 h. The procedure for the detection and calculation of residual biofilm was the same as described above.

*A. actinomycetemcomitans* cells were stained using a LIVE/DEAD Baclight Bacterial Viability Kit L7012 (Thermo Fisher Scientific, Waltham, MA, USA) by immersion in equal volumes of SYTO9 dye and PI dye for 10 min (The final working concentrations were 11 μM for SYTO 9 and 66 μM for PI). The biofilms were rinsed with PBS and examined under a confocal laser scanning microscope (LSM880, Zesis, Germany) (Qi et al., 2021). The lense used in the microscope is Plan-Apochromat 20×/0.8 M27 and the filters of SYTO and PI is 488 and 543nm, respectively. The COMSTAT computer program was used to analyze the structural organization of the microbial communities by quantifying three-dimensional biofilm image stacks.

### Quantitative real-time polymerase chain reaction (qRT-PCR) assay

*A. actinomycetemcomitans* was cultured with or without enzyme Est816, antibiotics or both for 48 hours. Total RNA was isolated using the RNeasy Mini Kit (Qiagen, Hilden, Germany). cDNA was synthesized from 200 ng of RNA using the Prime Script RT Reagent Kit (Takara, Kusatsu, Japan). The sequences of the PCR primers used in this study are listed in **Supplementary Table 1**. The PCR reaction parameters were as follows: initial denaturation step of 95°C for 30 seconds, and another 40 cycles of melting step at 95°C for 3 seconds, followed by 60°C for 30 seconds. The 2 ^−ΔΔCt^ method was used to analyze the relative expression levels of target genes.

### *In vivo* study

The biocompatibility of Est816 on Human gingival fibroblasts (HGF) was tested in advance of the *in vivo* rat experiments. HGF were procured from Anhui Hanjin Science and Technology Co. and utilized as experimental cells to assess cell morphology via immunofluorescence staining. The cells were treated with MINO (MIC), Est816 (12 U/mL), and a combination of MINO (MIC) and Est816 (12 U/mL) for three days. Untreated cells were used as a control group. Fibrillar actin (F-actin) structures were detected using standard TRITC-phalloidin staining (Sigma, St. Louis, MO, USA) (Zhu et al., 2023). Subsequently, the cells were examined under a fluorescence microscope.

The 36 Sprague-Dawley rats aged 6-8 weeks (weighing 180 – 200 g) used in this study were obtained from the Animal Experimental Center of Anhui Medical University (Anhui, China). The protocol for all experiments described herein adhered strictly to the ARRIVE and the Use of Laboratory Animals of the National Institutes of Health guidelines. This study was approved by the Animal Ethics Committee of the Anhui Medical University, China (Protocol No. LLSC20230821). Pluronic F127 (Sigma-Aldrich) serves as a topical drug delivery system that improves *in situ* drug adhesion without compromising drug efficacy or achieving stable and prolonged drug delivery (Almoshari et al., 2020). The rats were anesthetized, and silk ligatures were placed around the cervical part of the first upper molar. The rats were divided into six groups, each consisting of six animals: an untreated control group, a ligated control group, an experimental periodontitis group (0.15 ml of 1.0 × 10^6^ CFU/mL *A. actinomycetemcomitans* suspension every 4 days), an experimental periodontitis group treated with enzyme Est816 (a mixture of *A. actinomycetemcomitans* and 12 U/mL of enzyme Est816 with Pluronic F127 every 4 days), an experimental periodontitis group treated with MINO at the MIC, and an experimental periodontitis group treated with the combination of antibiotics and enzyme Est816.

### Micro-computed tomography (Micro-CT) analysis

After 8 weeks, the rats were sacrificed, and their maxillae were removed for further evaluation. Micro-CT scanning of the maxillae was performed using (Micro-CT; SkyScan, Kontich, Belgium), and the data were analyzed using the CT Analyzer software (version 1.15.4.0+; Skyscan). The area from the cemento-enamel junction (CEJ) to the alveolar bone crest (ABC) of the molar was defined as the vertical alveolar bone resorption.

### Histological and immunohistochemical analysis

The rat jaws were fixed in a 10% formaldehyde solution at 4°C for 1 week. After rinsing with distilled water, the samples were decalcified in 10% thylenediaminetetraaceticacid for 8 weeks. Subsequently, they were rinsed in PBS buffer for 12 hours, dehydrated, embedded, and cut into 3 – 4 μm sections. These sections were subjected to hematoxylin and eosin (H&E) staining and immunohistochemistry to detect matrix metalloproteinase-9 (MMP-9) expression in the periodontal tissues. The results were analyzed to assess the integrity and inflammatory response of the alveolar bone and cementum by histological observation using an optical microscope (OLYMPUS AX80, Olympus Co., Tokyo, Japan).

### Statistical analysis

The data were presented as the mean ± the standard error of the mean. One-way or two-way analysis of variance (ANOVA) followed by Bonferroni’s test were used to test for significance among groups. *P* values< 0.05 indicated statistical significance.

## Results

### Effect of added enzyme Est816 on the antimicrobial efficacy of antibiotics

**Figure 1:**
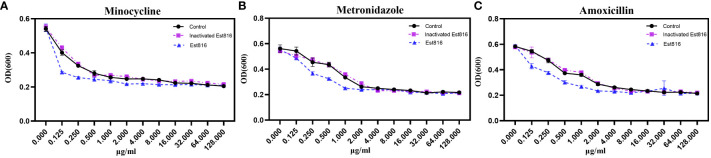
Minimum inhibitory concentration (MIC) results for *A. actinomycetemcomitans* of **(A)** Minocycline, **(B)** Metronidazole and **(C)** Amoxicillin. The antibiotic concentrations ranged from 0.125 to 128 μg/mL. For the inactivated enzyme group, the enzyme was inactivated by heating. The error bars in each panel represent the SD of triplicates.

In order to determine whether enzyme Est816 enhanced the sensitivity of *A. actinomycetemcomitans* to antibiotics, the MICs of MINO, MTZ, and AMX in the presence and absence of enzyme Est816 were assessed (**Figures 1A–C**). In this study, it was observed that inactivated enzyme Est816 did not have a significant impact on the MICs (**Figure 1**). The MIC of MINO when combined with enzyme Est816 was 0.25 μg/mL, while the MIC for the control and inactivated enzyme groups was 0.5 μg/mL (**Figure 1A**). Both MTZ and AMX exhibited a MIC of 4 μg/mL in the control and inactivated enzyme groups, whereas the MIC of the combination group was 2 μg/mL (**Figures 1B, C**). These findings indicate that the addition of enzyme Est816 synergizes with antibiotics to inhibit the growth of the growth of *A. actinomycetemcomitans*.

### Inhibition of *A. actinomycetemcomitans* biofilm formation by the combination of antibiotics with enzyme Est816

**Figure 2:**
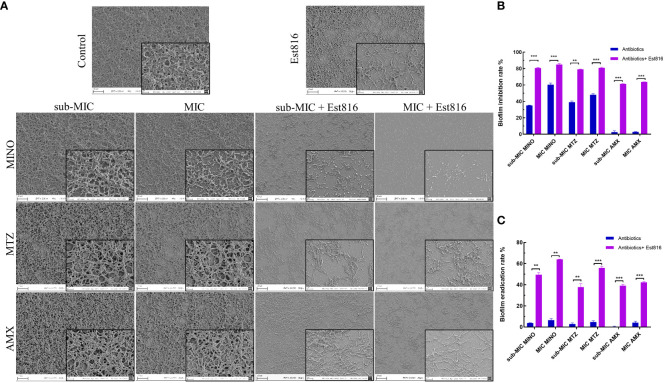
Morphology and biofilm formation of *A*. *actinomycetemcomitans*. **(A)** SEM revealed the effect of 12 U/mL lactonase Est816 with and without antibiotics on *A*. *actinomycetemcomitans* biofilm (original magnification × 1,000 and × 5,000). **(B)** Biofilm inhibition rate of minocycline (MINO), metronidazole (MTX) and amoxicillin (AMX) alone and in combination with enzyme Est816. **(C)** Biofilm eradication rate of MINO, MTZ, and AMX alone and in combination with enzyme Est816. (Compare within groups, ^**^ *P*<0.01, ^***^ *P*<0.001).

In the untreated group, the bacterial monolayer adhered to the slide surface and formed an overlapping structure of multiple layers (**Figure 2A**). However, in group Est816, the biofilm structures of *A. actinomycetemcomitans* collapsed, confirming that enzyme Est816 had a dispersing effect on biofilm formation. The combination of antibiotics with enzyme Est816 was significantly more synergistic than antibiotics alone in inhibiting biofilm formation. The biofilm grown in the presence of antibiotics and enzyme Est816 was evenly distributed in a single layer. Notably, a reduction in the biofilm biomass was observed even at sub-MIC concentrations of the synergistic antibiotics when combined with enzyme Et816. These results highlight the synergistic enhancement of the antibacterial effects of the antibiotics in the presence of enzyme Est816, effectively hindering *A. actinomycetemcomitans* biofilm formation.

Subsequently, crystal violet staining and quantitative analysis were used to determine the biofilm inhibition and eradication rates. The combined treatment exhibited superior inhibition of *A. actinomycetemcomitans* biofilm formation compared to the antibiotics alone (*p<* 0.01) (**Figure 2B**). When combined with enzyme Est816, each antibiotic effectively prevented biofilm formation at sub-MIC concentrations, demonstrating significant biofilm inhibition compared with its use alone (*p<* 0.01). AMX alone showed limited efficacy in removing biofilms; however, in combination with enzyme Est816, it significantly reduced biofilm formation. Disruption of mature biofilms by the combination of antibiotics with enzyme Est816 was also measured (**Figure 2C**). Notably, the three antibiotics showed weak activity at the sub-MIC and MIC values against mature *A. actinomycetemcomitans* biofilms. The synergistic effect of the enzyme Est816 significantly increased the eradication rate of antibiotic treatment compared to antibiotics alone (*p<* 0.01).

**Figure 3:**
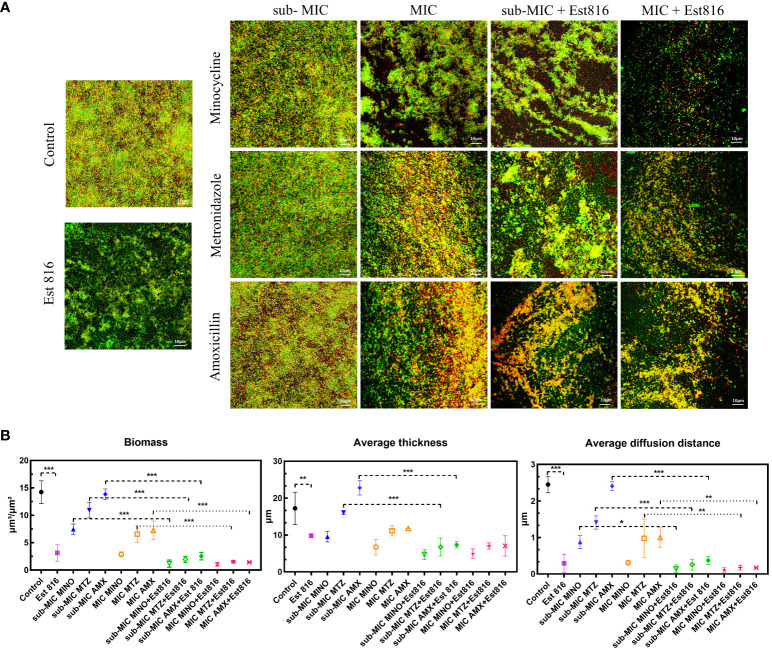
Inhibition of *A*. *actinomycetemcomitans* biofilm by enzyme Est816 and the antibiotics (1:1 ratio). **(A)** Confocal laser scanning microscopy (CLSM) analysis of *A. actinomycetemcomitans* biofilm. **(B)** Biofilm biomass, average thickness, and average diffusion distance quantified by COMSTAT software. The error bars in each panel represent the SD of triplicates. *P<0.05, **P<0.01, ***P<0.001.

The ability of the synergia of the antibiotics and enzyme Est816 to disperse pre-formed biofilms was further explored using CLSM. There was a significant difference in the overall structure and thickness of biofilms between the combination and control groups (**Figure 3A**). The biofilms in the control group were dense and thick. After treatment with the antibiotics and enzyme Est816, the overall structure of the biofilm was disrupted. Compared with the antibiotic-only group, the enzyme Est816-antibiotic combination group showed a greater decrease in cell density, indicating that enzyme Est816 enhanced the inhibition of biofilm formation. For a comprehensive assessment of the impact of combination agents on bacterial biofilms, CLSM imaging with COMSTAT analysis was employed to analyze the architecture of the biofilms. This allowed for the visualization and characterization of the *in-vitro* biofilms. After 48 hours of treatment with or without enzyme Est816, the biomass and average thickness of the biofilm decreased at varying concentrations of antibiotics. The changes in the trend observed in each group were consistent with the results of biofilm reduction in crystal violet biofilm quantification (**Figure 3B**). When antibiotics (sub-MIC) were combined with enzyme Est816, the average thickness, biofilm biomass, and diffusion distance of *A. actinomycetemcomitans* biofilm were significantly lower and statistically different from those treated with antibiotics alone, except for MINO (*p<* 0.01). Furthermore, regardless of the sub-MIC or MIC concentration of the antibiotics, there was no statistically significant difference in the three quantitative measurements of biofilms in the combined group with enzyme Est816 (*p* > 0.05).

### Effect of antibiotics combined with enzyme Est816 on virulence expression

To understand the mechanisms underlying antibacterial and anti-biofilm activities, we conducted qRT-PCR on two genes responsible for pathogenicity and biofilm formation.

**Figure 4:**
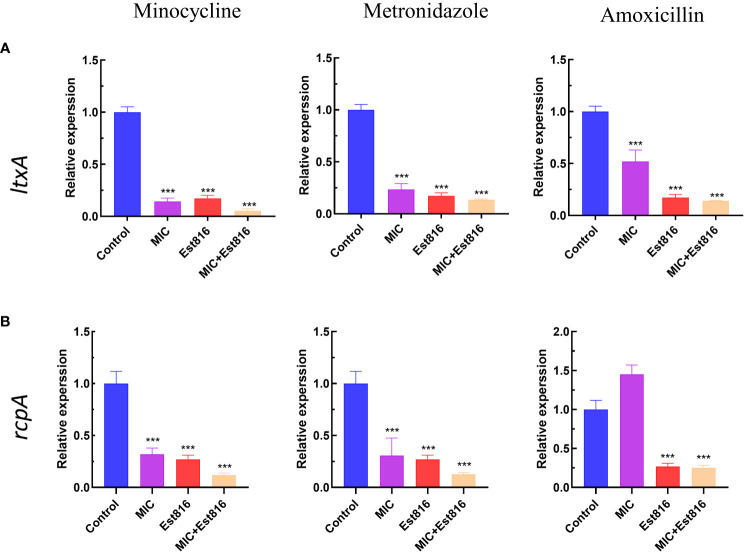
Effect of enzyme Est816 and the three antibiotics on the expression of virulence genes and exopolysaccharides. Real-time polymerase chain reaction analysis of the effect of enzyme Est816 and the antibiotics on the expression of **(A)** *ltxA* and **(B)** *rcpA*. (Compared with the control group, ^***^ *P*<0.001).

Compared to control group, the combination of antibiotics with enzyme Est816 showed a strong inhibitory effect on the expression of virulence factors *ltxA* and *rcpA* (*p<* 0.01) (**Figure 4**). The increase in *rcpA* caused by AMX indicates a different mechanism of action for antibiotics on bacteria. The decrease in *rcpA* expression observed during combination therapy is a result of the Est816 effect.

### Biocompatibility of enzyme Est816

**Figure 5:**
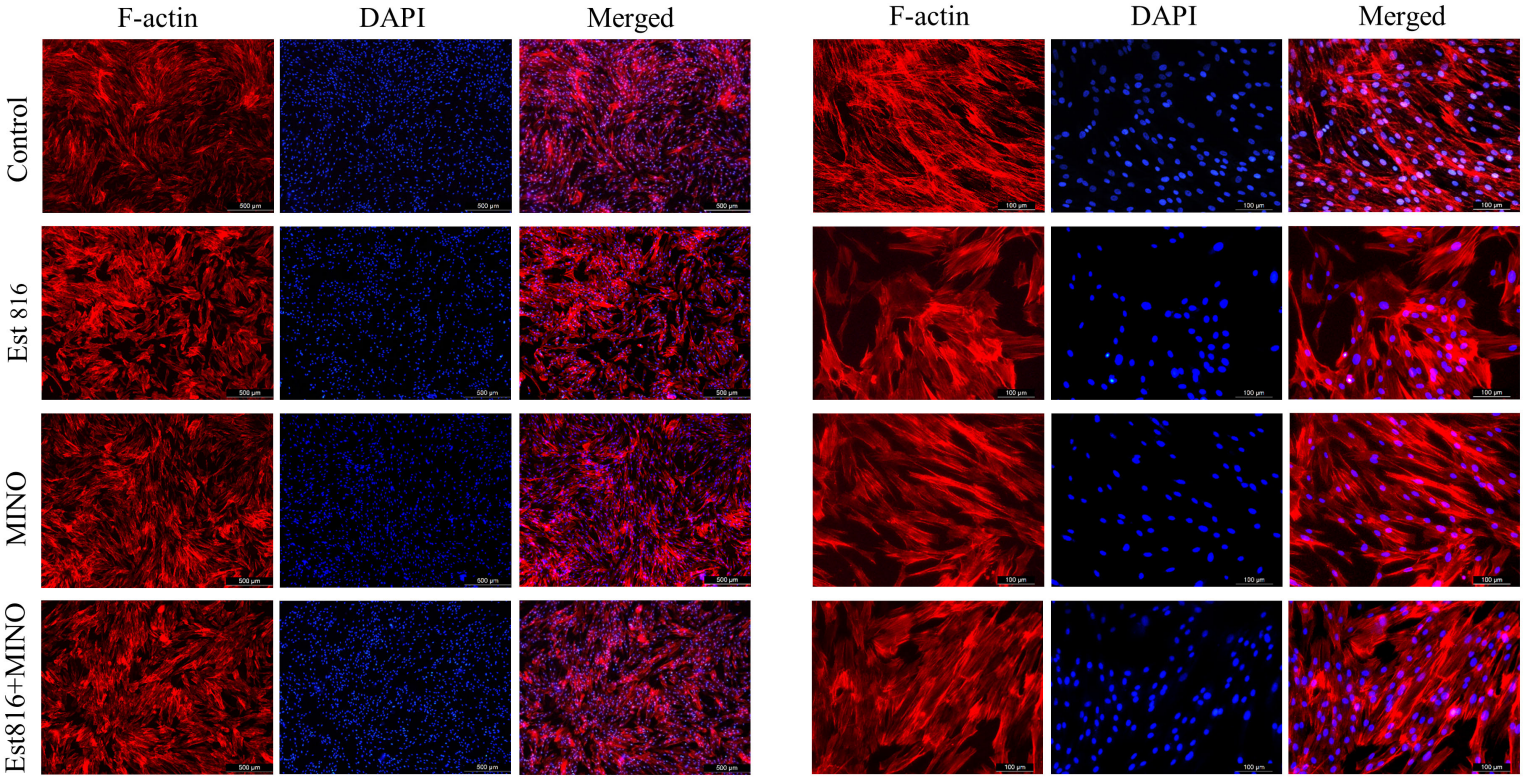
Typical appearance of human gingival fibroblast (HGF) cells with blue DAPI and F-actin staining. TRITC-phalloidin fluorescence staining of cells after Ese816, MINO, and combined treated with HGF cells for 72 hours.

Immunofluorescence staining of HGF cells was performed to determine compatibility with enzyme Est816. As shown in **Figure 5**, after 3 days of incubation, there was no significant difference in cell proliferation between the control group and group Est816. In addition, no changes in DAPI fluorescence intensity were detected in any of the study groups compared with the control group. The results showed that cells cultured with MINO at the MIC and enzyme Est816 (12 U/mL) did not exhibit significant morphological or nuclear damage compared with the control group. Additionally, structural reorganization of the actin cytoskeleton was not detected at the end of cultivation with enzyme Est816, indicating acceptable biocompatibility.

### Therapeutic effect of the combination of enzyme Est816 and MINO on periodontitis in rats

**Figure 6:**
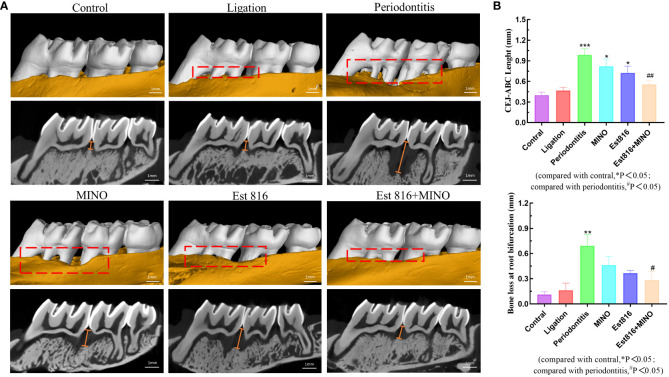
Bone resorption of maxilla was assessed by micro-computed tomography. **(A)** The red area indicates the site of the bone loss, and the yellow line indicates the crest height between the cementoenamel junction (CEJ) and the alveolar bone crest (ABC). **(B)** Distance of alveolar crest absorption and bone loss of the root bifurcation in the control, periodontitis, ligation, MINO, enzyme Est816, and mixed groups for 2 months, respectively. Compared with the control group, *P<0.05, **P<0.01, ***P<0.001; compared with the periodontitis group, ^#^ *P*<0.05, ^##^ *P*<0.01.

**Figure 7:**
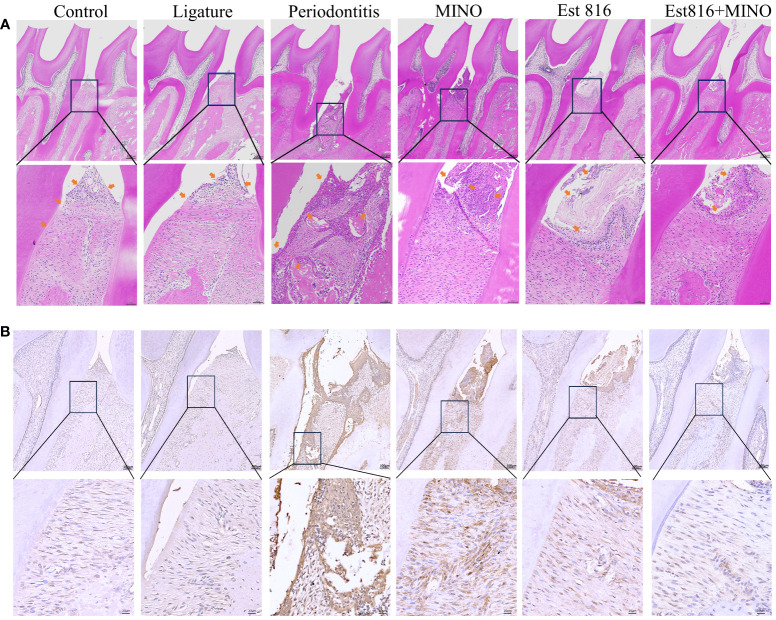
Histological staining of the periodontal tissues in rats. **(A)** Hematoxylin and eosin staining (magnification ×5 and ×20); yellow-headed arrow indicates the inflammation of the gingival tissue. **(B)** Immunohistochemical staining for matrix matalloproteinsase 9 in periodontal tissue (magnification ×10 and ×40).

Micro-CT analysis was performed to assess the therapeutic effects of the combination of enzyme Est816 and MINO on periodontitis *in vivo*. Bone resorption was minimal in the ligation group, whereas bone loss was most pronounced in the periodontitis group, as was evident in the root furcation area and alveolar septum (**Figure 6A**). To accurately assess alveolar bone resorption, we measured the distance from the CEJ to the ABC and the loss of bone in the bifurcation area. Compared to the control group, the experimental periodontitis group showed a significant increase in periodontal bone resorption (*p<* 0.01), while the MINO and enzyme Est816 groups showed a decrease in bone resorption compared to the experimental periodontitis group (**Figure 6B**). Compared with periodontitis, the combination of Est816 and MINO resulted in a significant reduction in bone loss (*p<* 0.01). H&E staining showed that the gingival epithelium and alveolar bone between the first and second molars of rats in the control group were both present and healthy (**Figure 7A**). In the ligated control group, the gingival epithelium was detached from the tooth at the ligation site (arrow), and the alveolar bone was only slightly lower than that in the control group. In the periodontitis group, sequestrum formation and deep periodontal pockets were observed under the microscope. In the antibiotic group, loss of gingival tissue and an increase in the number of inflammatory cells were observed, while in the enzyme group, the gingival tissue was partially destroyed, and alveolar bone was resorbed. The combined Est816 intervention showed no evidence of periodontal pockets. Thus, alveolar bone levels were essentially unaffected, and only epithelial damage caused by mechanical stimulation was observed. Immunohistochemical staining for MMP-9 showed no obvious inflammatory reactions in the control, ligation, or combined groups (**Figure 7B**). In addition, the group that received antibiotics and enzyme Est816 showed reduced expression of MMP-9 compared to the periodontitis group; however, the individual effects of enzyme Est816 and MMP-9 were far less pronounced than their combined effect. When taken together, these results indicate that the components of the combination treatment had a marked synergistic effect in treating periodontitis, even in the absence of debridement or irrigation.

## Discussion

Local antibiotics, such as MINO, MTZ, and AMX, have been used to prevent the recurrence of periodontal disease after mechanical debridement. MINO has broad-spectrum activity against aerobic, anaerobic, Gram-negative, and Gram-positive bacteria and has the advantage of excellent tissue distribution. Inubushi et al. tested the antibacterial susceptibility of standard (19 isolates) and clinical (90 isolates) aerobic and anaerobic oral bacteria to various antibiotics (Inubushi and Liang, 2020). Their results, like those presented here, indicate that MINO has relatively strong antibacterial activity against *A. actinomycetemcomitans* compared with other antibiotics. The combination of MTZ and AMX has been reported to be particularly effective in the treatment of *A. actinomycetemcomitans*-associated periodontitis (Sgolastra et al., 2012; Zandbergen et al., 2013; Feres et al., 2018). Penicillins, especially AMX, appear to be highly effective against most periodontal pathogens *in vitro* (Gordon and Walker, 1993). MTZ is a chemotherapeutic agent with broad-spectrum activity against anaerobic bacteria (Greenstein, 1993). The combination of multiple antibiotics has been used in clinical practice to treat drug-resistant bacterial infections (Leu et al., 2014); however, it remains difficult to prevent the emergence of new drug-resistant strains. Moreover, bacteria in biofilms are more resistant to antimicrobial agents than planktonic cells because of their extracellular matrix content. Therefore, biofilm disruption may have a significant synergistic effect with antibiotics in the clinical treatment of periodontitis.

Quorum-quenching offer a promising strategy to mitigate bacterial virulence, demonstrating advantages in controlling the emergence and spread of antibiotic-resistant phenotypes compared to conventional antibiotics. In this study, the MIC results showed that enzyme Est816 therapy increased the susceptibility of *A. actinomycetemcomitans* to the three antibiotics tested, reducing it by half compared to the untreated group after combination with enzyme Est816. Notably, MINO showed the most rapid inhibition when combined with enzyme Est816. Several limitations related to antibiotic resistance have been reported (Veloo et al., 2012). *Porphyromonas gingivalis* biofilm cells are resistant to antibiotics at concentrations 100 times the MIC for planktonic organisms (Eick et al., 2004). Thus, the traditional treatments for periodontitis may become less effective as more bacteria become resistant to antibiotics (Slots and Rams, 1990). However, in the presence of enzyme Est816, biofilm growth can be controlled using lower concentrations of antimicrobial agents. Other researchers have reported that *N*-acylhomoserine lactonase improved the antibiotic susceptibility of *Pseudomonas aeruginosa* (Zhang et al., 2023). In addition, a membrane-disrupting agent has been used to treat infections of joint prostheses and demonstrates a powerful anti-membrane effect when combined with antibiotics (Li et al., 2022). Likewise, lactonase Est816, an antibiofilm agent, significantly increased the susceptibility of *A. actinomycetemcomitans* to various antibiotics. Based on our previous studies, we confirmed that enzyme Est816 had a significant effect on inhibiting biofilms (Zhao et al., 2024). In this study, we investigated the synergistic effect of antibiotics and enzyme Est816 on biofilm inhibition, providing new support for further research on periodontal biofilm infections.

Morphological observations revealed that, at their MICs, the individual antibiotics exhibited minimal impact on the ultrastructure of *A. actinomycetemcomitans* biofilm. However, pretreatment with these antibiotics in combination with enzyme Est816 demonstrated inhibition of biofilm formation, with the combination with MTZ proving especially effective. Quorum-quenching enzymes can modify bacterial properties to prevent the initial attachment, thereby reducing biofilm formation. This enzymatic intervention leads to the disaggregation of densely packed bacteria, facilitating increased diffusion of antibiotics and resulting in a more pronounced reduction in biofilm formation compared to when antibiotics alone are used. Consistent with the SEM results, crystal staining and measurement of the minimal biofilm eradication rate showed that enzymatic intervention not only inhibited biofilm formation but also disrupted the integrity of mature biofilms.

Compared to the combination of Est816 and MTZ or AMX, the combination of this AHL-degrading enzyme with MINO was markedly more effective in terms of dead cells, total biomass, average thickness of the biofilm, and average diffusion distance of the antibiotics. This result may be attributed to the mechanism by which tetracycline inhibits the adhesion and co-aggregation of Gram-negative bacteria at sublethal concentrations. Previous studies suggested that MINO is highly effective against *A. actinomycetemcomitans* bacterium (Slots et al., 1980). We hypothesized that this may be related to the bactericidal mechanism by which MINO inhibits early protein synthesis. Previous studies have demonstrated the inhibitory effect of MINO on biofilm growth. These results demonstrate that synergy therapy is more effective than antibiotics alone in terms of antibacterial activity.

Certain virulence factors play crucial roles in the initial stages of biofilm formation and maturation, and their regulation is controlled by QS. Therefore, quorum-quenching enzymes are important targets for the development of anti-biofilm drugs. Rough colony protein A (RcpA) is important for the colonization and biofilm formation of *A. actinomycetemcomitans* (Rad et al., 2019). *rcpA* is involved in the secretion of assembled fimbriae onto the surface of microorganisms (Pourhajibagher et al., 2017). Leukotoxin is a potent virulence factor that induces an imbalance in the host inflammatory response (Johansson, 2011), and *in vitro* studies have shown that it induces neutrophil degranulation and macrophage death, ultimately leading to the activation of the immune response (Fives-Taylor et al., 1999). In this study, enzyme Est816 reduced the expression of *ltxA* and *rcpA*, thereby reducing the resistance of cell membranes to the external environment and increasing the effectiveness of antibiotics in killing bacteria. These results suggest that the combination strategy eradicated the biofilm and facilitated the diffusion of antibiotics into deep cell layers. Unlike AHL analogs or antibiotics, AHL lactonases specifically target AHLs secreted by bacteria for hydrolysis without affecting mammalian cells (Wang et al., 2004). The results of the cytotoxicity experiments confirmed that Est816 had a negligible impact on the morphology of HGF cells.

Based on the finding of the powerful synergistic effect of MINO with enzyme Est816, this combination strategy was used to treat periodontitis in rats. Even in the absence of debridement and irrigation, the local application of enzyme Est816 or MINO alone resulted in a slight reduction in bone loss; however, the combination treatment was highly effective in reducing bone loss and inflammatory damage. *A. actinomycetemcomitans* can efficiently migrate through the gingival epithelium, inhibit bone collagen synthesis, and induce osteoblast apoptosis, thereby directly accelerating the progression of periodontitis (Fives-Taylor et al., 1999; Herbert et al., 2016). The enhanced antibacterial ability of antibiotics inhibit *A. actinomycetemcomitans*, while *A. actinomycetemcomitans* employs virulence factors, such as *ltxA*, which protects it from phagocytosis and induces a pro-inflammatory response (Oscarsson et al., 2020). Combination therapy decreases the release of virulence factors and MMP-9, thus indirectly restoring the imbalance in the host inflammatory response and resulting in a synergistic effect against periodontitis.

## Conclusion

This study demonstrated that the biofilm-targeting enzyme Est816 can serve as an adjunct therapy in combination with antibiotics to inhibit biofilm formation and enhance antibacterial activity, ultimately eliciting a significant therapeutic effect on periodontitis. The fact that periodontitis is associated with dysbiosis of the subgingival flora not only adds to the complexity of the oral microbiota, but also has implications for the bacterial-host immune response, which merits further investigation.

## Data availability statement

The raw data supporting the conclusions of this article will be made available by the authors, without undue reservation.

## Ethics statement

The animal study was approved by the animal ethics committee of Anhui Medical University, China. The study was conducted in accordance with the local legislation and institutional requirements.

## Author contributions

JW: Conceptualization, Data curation, Investigation, Writing – original draft, Writing – review & editing. TJ: Data curation, Investigation, Writing – original draft, Writing – review & editing. LG: Formal analysis, Methodology, Writing – original draft, Writing – review & editing. WS: Data curation, Software, Writing – original draft, Writing – review & editing. QW: Data curation, Software, Writing – original draft, Writing – review & editing. HZ: Writing – original draft, Writing – review & editing, Data curation, Software. JZ: Project administration, Resources, Writing – original draft, Writing – review & editing.
