# Supplementary material for: Quorum-quenching enzyme Est816 assisted antibiotics against periodontitis induced by Aggregatibacter actinomycetemcomitans in rats
Source: Front Cell Infect Microbiol. 2024 May 8;14:1368684. doi: 10.3389/fcimb.2024.1368684 (PMC11109752; doi:10.3389/fcimb.2024.1368684)
Supplement: Supplementary file 1 [file DataSheet_1.docx]

**Supporting information**

**Quorum-quenching Enzyme Est816 Assisted Antibiotics against Periodontitis Induced by *Aggregatibacter actinomycetemcomitans* in Rats**

**MATERIALS AND METHODS**

**Biofilm assessment**

**Determination of the biofilm inhibition rate and biofilm eradication rate**

For crystal violet semiquantitative biofilm assay, the 500 μL *A. actinomycetemcomitans* suspension (1 × 10^6^CFU/mL) was added to 24-well plates, and 500 μL PBS, antibiotics (sub-MIC or MIC), and Est816 were added to each well individually to co-culture with the biofilm. After incubation for 48 hours at 37 ℃ under microaerophilic conditions, the biofilm was fixed with 400 μL paraformaldehyde solution for 15 minutes. The solution was then removed, and the biofilms were stained with crystal violet solution (0.1%, 300 μL) for 20 min. The biofilms were then washed three times, and the crystal violet stain was dissolved with 400 μL of 95% ethanol. The OD values of a semiquantitative analysis of the biofilm were detected at 570 nm on a microplate reader. For determination of the biofilm inhibition rate and biofilm eradication rate, the percentages of biofilm inhibition/reduction were calculated as follows: Biofilm Reduction% = $=\frac{\mathrm{Abs}\mathrm{Control} - Abs \mathrm{Sample}}{\mathrm{Abs}\mathrm{Control}}$× 100%. The biofilm formed in the 96-well plate was incubated at 37 ◦C for 48 hours to obtain a mature biofilm. Eradication was performed by adding antibiotics at concentrations of sub-MIC, MIC with or without Est816 to the mature biofilm and incubated at 37 ◦C for another 24 h. The procedure for detection and calculation of residual biofilm was the same as described above.

**Effects of Est816 against the** **progression of periodontitis in rats**

**Histopathological and Immunohistochemical Analysis**

At 8 weeks after decalcification, the maxillae were decalcified with 10% EDTA, and the tissues from the first to the second molars were imbedded in paraffin to obtain 4-μm continuous slices in the sagittal plane prepared for hematoxylin and eosin (HE) staining and immunohistochemistry staining. H&E staining (5 specimens/group) was performed to assess the integrity and inflammatory response of alveolar bone and cementum under histological observation by an optical microscope (OLYMPUS AX80, Olympus Co., Tokyo, Japan).

**Histopathological Assay**

H&E staining was performed to assess the integrity and inflammatory response of alveolar bone and cementum under histological observation by an optical microscope (OLYMPUS AX80, Olympus Co., Tokyo, Japan).

**Immunohistochemical Analysis**

To evaluate the levels of matrix metalloproteinase-9 (MMP-9), in periodontal tissue from each group, each tissue slice (5 specimens/group) was deparaffinized and rehydrated and then treated with 0.05% trypsin to extract antigen. After washing with PBS, the sections were quenched with 3% hydrogen peroxide for endogenous peroxidase blockade and incubated with primary antibodies (Santa Cruz Biotechnology, INTERPRISE, Brazil): MMP-9, 1:400m overnight at 4 °C for 2 h. Then, slices were washed and incubated with a streptavidin-HRP-conjugated secondary antibody (Biocare Medical, CA, USA) for 30 min. A colorimetric-based detection kit (Biocare Medical, CA, USA) was used to visualize the immunoreactivity to MMP-9.

**Supplementary Figures and Tables**

**Supplementary** **Table 1.**

**Table 1.** Polymerase chain reaction primers of *A. actinomycetemcomitans.*

| **Primer** | **Forward (5’-3’)** | **Reverse (5’-3’)** |
| --- | --- | --- |
| 16sRNA | AAGCACCGGCTAACTCCGT | TTCCGATTAACGCTCGCAC |
| *ltx*A | TCGCGAATCAGCTCGCCG | GCTTTGCAAGCTCCTCACC |
| *rcp*A | TGGGCATTAACTGGAGCCAC | ATCCACCTCCGAAACCGAAG |
